# Evaluation of Multi‐Breed Genomic Prediction Models for Carcass and Reproductive Traits in Zebu Beef Cattle

**DOI:** 10.1002/age.70188

**Published:** 2026-08-20

**Authors:** Gabriel Gubiani, Larissa Temp, Marisol Londoño‐Gil, Miller Teodoro, Eduarda Oliveira, Ignacio Aguilar, Matias Bermann, Daniela Lourenco, Angelica S. C. Pereira, Fernando Baldi

**Affiliations:** ^1^ Department of Veterinary Medicine, Faculty of Animal Science and Food Engineering University of São Paulo Pirassununga Brazil; ^2^ Department of Animal Science, School of Agricultural and Veterinary Sciences São Paulo State University (UNESP) Jaboticabal Brazil; ^3^ Instituto Nacional de Investigación Agropecuaria (INIA) Canelones Uruguay; ^4^ Department of Animal and Dairy Science University of Georgia Athens Georgia USA; ^5^ Department of Animal Nutrition and Production, Faculty of Veterinary Medicine and Animal Science University of São Paulo Pirassununga Brazil

**Keywords:** *Bos indicus*, genomic relationship, metafounders, multi‐breed models, ssGBLUP

## Abstract

Genomic prediction in beef cattle is particularly challenging in breeds with limited phenotypic and genotypic data. In this context, multi‐breed methodologies that integrate information from genetically related populations have emerged as a promising strategy to improve prediction accuracy and calibration under data‐scarce conditions. This study evaluated different genomic prediction approaches in a multi‐breed population of Zebu cattle, including Nellore, Guzerat, Brahman, and Tabapua, using 653 785 phenotypic records, 190 865 genotypic records, and 3 681 158 pedigree records. The traits analyzed were rib eye area (REA), rump fat thickness (RFT), age at first calving (AFC), and accumulated productivity (ACP). Genomic estimated breeding values were obtained using the single‐step GBLUP method under four models: single‐breed (G_SB_), standard multi‐breed (G_0_), metafounders (MF), and an adjusted genomic relationship matrix (AGR). Model performance was assessed using the linear regression method, which compares predictions from complete and partial datasets to estimate accuracy, bias, and dispersion. Multi‐breed models, particularly MF and AGR, produced higher accuracy than the single‐breed approach in several analyses for underrepresented breeds such as Guzerat, Brahman, and Tabapua, especially for carcass and reproductive‐related traits. For example, in Guzerat, REA accuracy increased from 0.44 with G_SB_ to 0.62 with AGR, while in Tabapua, ACP accuracy improved substantially from 0.23 with G_SB_ to 0.51 with AGR. These results highlight the importance of leveraging genetically related breeds and well‐structured reference populations to improve the reliability of genomic predictions in Zebu cattle under limited data conditions.

## 1 Introduction

The accuracy of genomic prediction is closely linked to the size and quality of the reference population, as these factors determine the power to detect associations between genetic markers and phenotypic traits (Hayes et al. 2009; Lee et al. 2020; Al‐Khudhair et al. 2021; Rodriguez Neira et al. 2022). In breeds with limited data availability, the high costs associated with phenotypic and genotypic data collection hinder the development of robust prediction models (Hayes et al. 2009; Liu et al. 2019). In this scenario, integrating genomic and phenotypic data from breeds with larger and better‐characterized datasets represents a promising strategy to enhance prediction accuracy in data‐scarce populations (Hayes and Goddard 2008; Calus et al. 2018).

However, the absence of genotypes from founding animals limits the construction of the genomic relationship matrix (**G**) across populations, primarily due to the lack of baseline allele frequency information (Legarra et al. 2015). One strategy to address this limitation is the use of multi‐breed populations, particularly when genetically related breeds are combined (Cesarani et al. 2022). This approach has proven effective in forming metapopulations, increasing the robustness of genomic analyses, and improving prediction accuracy in underrepresented groups (Raymond et al. 2018). The combination of breeds with shared ancestry tends to enhance predictive performance, whereas the inclusion of genetically distant populations often yields more modest gains (Pryce et al. 2011; Brøndum et al. 2011; Hozé et al. 2014).

Among Zebu breeds, Nellore stands out for its high adaptability to tropical environments, favorable growth performance, and advantageous genetic aspects (Barreto et al. 2024). As a result, it plays a central role in breeding programs tailored to tropical systems (Paschal et al. 1995; Favero et al. 2019; Ogunbawo et al. 2024). In contrast, breeds such as Guzerat, Brahman, and Tabapua face challenges in genomic evaluations due to the limited availability of pedigree, phenotypic, and genotypic data. In this context, multi‐breed strategies offer a valuable opportunity to improve prediction accuracy, as the shared ancestry among these breeds facilitates the identification of common alleles that can be leveraged in genomic models (Porto‐Neto et al. 2013; Decker et al. 2014).

Among genomic prediction methodologies, the single‐step genomic best linear unbiased prediction model (ssGBLUP) stands out for its ability to integrate pedigree, phenotypic, and genomic data into a unified analysis (Legarra et al. 2009, 2015; Christensen and Lund 2010). The robustness of ssGBLUP is grounded in the principles of GBLUP, whose accuracy largely depends on the size of the genotyped reference population (Goddard et al. 2009; Pszczola et al. 2012; Zhang et al. 2024). By combining all sources of information, ssGBLUP has demonstrated promising results, particularly in multi‐breed populations, enhancing prediction accuracy for breeds with limited data (Londoño‐Gil et al. 2025).

The choice of a multi‐breed approach should consider both the purpose of evaluation and the population structure. The metafounders (MF) approach represents ancestral populations and enables modeling of genomic relationships among founders from different origins, facilitating information flow between breeds, enhancing the connectivity of the **A**_22_ matrix, and improving compatibility between the **G** and **A**_22_ matrices (Legarra et al. 2015). The adjusted genomic relationship (AGR) model modifies the **G** based on breed‐specific allele frequencies, thereby preserving differences among breeds while maintaining the kinship matrix of non‐genotyped individuals (Makgahlela et al. 2013, 2014; Lourenco et al. 2016). In contrast, the G_0_ model assumes a single genetic base for the entire genotyped population, applying the standard **G**, which considers the same (current) allele frequencies across all populations. This assumption can enlarge the genomic training population and improve predictive accuracy (Hayes and Goddard 2008), particularly in joint evaluations across breeds.

Traits such as accumulated productivity (ACP) and age at first calving (AFC), along with rib eye area (REA) and rump fat thickness (RFT), are key targets in beef cattle breeding programs. However, reproductive traits are recorded exclusively in females, which limits the volume of available data (Paterno et al. 2017; Bonamy et al. 2019), while carcass traits depend on costly procedures and specialized equipment (Gordo et al. 2012). This scarcity of records reduces the accuracy of genomic predictions. In this context, multi‐breed evaluation approaches represent a viable alternative to expand the reference population and overcome these limitations, particularly in less‐represented breeds.

Although these approaches have been evaluated previously, direct comparisons of alternative genetic‐base representations across data‐rich and data‐limited *Bos indicus* breeds remain scarce, particularly when carcass and female‐limited reproductive traits are assessed under the same validation framework. Given this context, the present study aims to evaluate different genomic prediction strategies in a multi‐breed population composed of Nellore, Guzerat, Brahman, and Tabapua animals, focusing on carcass traits such as rib eye area (REA) and rump fat thickness (RFT), and reproductive traits such as age at first calving (AFC) and accumulated productivity (ACP). The objective was to compare the effects of G0, AGR, and MF on the accuracy, bias, and dispersion of genomic predictions for REA, RFT, AFC, and ACP in breeds with different data availability.

## 2 Materials and Methods

### 2.1 Ethics Statement

This study was exempt from evaluation by the Animal Ethics Committee (CEUA), as established by Law No. 11794 of 08/10/2008 and Normative Resolution No. 51 of 05/19/2021 from the National Council for Animal Experimentation Control (CONCEA) because the data were obtained from an existing database.

### 2.2 Phenotypic and Pedigree Data

The database used in this study was provided by the animal breeding programs coordinated by the National Association of Breeders and Researchers (ANCP), based in Ribeirão Preto, São Paulo, Brazil. A total of 653 785 phenotypic records and 190 865 genomic records were analyzed for the traits rib eye area (REA), rump fat thickness (RFT), age at first calving (AFC), and accumulated productivity (ACP) in Nellore, Guzerat, Brahman, and Tabapua cattle (Table 1). The dataset includes animals born between 2002 and 2021, originating from 40 farms located across the Central West, Southeast, North, and Northeast regions of Brazil. The pedigree file contained information on 3 681 158 animals. Integration of phenotypic, genotypic, and pedigree data for estimating genomic breeding values was performed using the ssGBLUP model.

**TABLE 1:** Descriptive statistics for rib eye area (REA), rump fat thickness (RFT), age at first calving (AFC), and accumulated productivity (ACP) in Nellore, Guzerat, Brahman, and Tabapua breeds.

Trait		Breed				Total
		Nellore	Guzerat	Brahman	Tabapua	
REA (cm^2^)	*N*	152 760	4999	4077	1648	163 484
	Mean	60.22	60.37	58.69	55.11	58.60
	SD	11.98	11.40	13.85	10.97	12.05
	NG	51 733	1532	4370	732	58 367
	NCG	4906	1131	640	183	6860
RFT (mm)	*N*	152 417	4996	4061	1643	163 117
	Mean	5.12	4.89	4.15	3.11	4.32
	SD	2.65	2.54	2.34	1.20	2.18
	NG	51 671	1532	4370	732	58 305
	NCG	4893	1131	640	183	6847
AFC (months)	*N*	176 921	12 555	38 073	7779	235 328
	Mean	32.58	37.26	36.38	37.44	35.92
	SD	6.67	6.07	4.60	5.84	5.80
	NG	49 765	1531	4369	731	56 396
	NCG	2350	1398	1680	646	6074
ACP (kg)	*N*	66 600	4734	18 483	2033	91 850
	Mean	161.32	132.62	137.83	145.68	144.36
	SD	33.67	31.18	25.83	30.19	30.22
	NG	11 166	1531	4369	731	17 797
	NCG	1155	778	754	274	2961

Abbreviations: *N*, phenotypic number; NCG, number of contemporary groups; NG, number of genotyped animals; SD, standard deviation.

The REA and RFT traits were measured in animals between 15 and 18 months of age, following the protocols established by the Ultrasound Guidelines Council and the Beef Improvement Federation (Hohenboken 2002). Images were captured from the *Longissimus dorsi* muscle, located between the 12th and 13th ribs, and from the rump region to assess subcutaneous fat thickness. Scanning and image analysis were conducted by technicians accredited by ANCP, strictly adhering to standardized procedures to ensure measurement accuracy and reproducibility.

The AFC trait was determined from the reproductive history of each female, based on the recorded date of birth and the date of first calving on the farm, serving as an indicator of reproductive efficiency. The ACP trait was calculated as the total weight of weaned calves produced per cow per year, considering AFC, calving intervals, and the duration of the female's stay in the herd, as described by Lôbo et al. (2000). These data, extracted from routine production records, reflect the cows' productive efficiency and their contribution to the overall herd performance.

### 2.3 Genotypic Data

The genomic dataset (Table 1) included genotypic information for all evaluated traits. Animals were genotyped using the medium‐density CLARIFIDE Nellore 3.0 panel, which contains approximately 30 000 SNP markers. Genotypes were imputed to the higher‐density CLARIFIDE Nellore 4.0 panel (~70 000 markers) using FIMPUTE 2.2 software (Sargolzaei et al. 2014). Imputation accuracy reached 98.5%, with 97.9% of genotypes correctly imputed, as reported by Carvalheiro et al. (2014). Quality control of SNP data was performed with the preGSF90 software from the BLUPF90 family of programs (Aguilar et al. 2014). Markers with a call rate lower than 0.90, minor allele frequency (MAF) below 0.05, Mendelian conflicts exceeding 0.01, and monomorphic SNPs were excluded. In addition, samples with a call rate below 0.90 were removed from the dataset. After quality control, 36 045, 36 039, 36 469, and 37 887 SNPs were retained in the REA, RFT, AFC, and ACP datasets, respectively.

### 2.4 Genomic Prediction Scenarios

Univariate analyses incorporating pedigree, phenotypic, and genomic information were performed. Contemporary groups (CG) were defined by combining the fixed effects of farm, year, season of birth, and management group during the rearing phase for all traits evaluated. Additionally, sex was included as a fixed effect in the CG formation only for carcass traits (REA and RFT), since AFC and ACP are recorded exclusively in females. CG with fewer than four observations, as well as phenotypic records deviating more than ±3 standard deviations from the mean, were excluded from the analysis. The general animal model used was:

(1) y=Xβ+Zu+e

where ***y*** is the vector of phenotypic records, β is the vector of fixed effects (e.g., CG and dam age at calving for all traits). For REA and RFT traits, animal age at the time of measurement was also included as a covariate, modeled with both linear and quadratic effects. ***X*** is the incidence matrix associating β with ***y***, u is the vector of direct additive genetic effects, Z is the incidence matrix associating u with ***y***, and ***e*** is the vector of residual effects.

Based on this model, variance components were estimated without the inclusion of genomic information using the BLUPF90+ software (Misztal et al. 2014; Lourenco et al. 2022). Analyses were performed separately for each breed and using a combined multi‐breed dataset. The resulting additive genetic and residual variance components were used as scaling parameters in all subsequent genomic prediction analyses (Table 2).

**TABLE 2:** Additive genetic variance, residual variance, and heritability estimates for rib eye area (REA), rump fat thickness (RFT), age at first calving (AFC), and accumulated productivity (ACP) in breed‐specific and combined multi‐breed analyses.

Trait	Population	σ^2^_a_	σ^2^_e_	h^2^ ± SD
REA	Nellore	14.54	26.11	0.36 ± 0.01
REA	Guzerat	15.42	31.88	0.33 ± 0.05
REA	Brahman	16.37	30.09	0.35 ± 0.06
REA	Tabapua	11.15	22.51	0.33 ± 0.08
REA	Multi‐breed	14.65	26.26	0.36 ± 0.01
RFT	Nellore	0.79	1.48	0.35 ± 0.01
RFT	Guzerat	0.71	1.34	0.35 ± 0.05
RFT	Brahman	0.55	0.77	0.42 ± 0.06
RFT	Tabapua	0.24	0.50	0.32 ± 0.08
RFT	Multi‐breed	0.76	1.47	0.34 ± 0.01
AFC	Nellore	1.17	20.09	0.06 ± 0.004
AFC	Guzerat	2.35	21.00	0.10 ± 0.02
AFC	Brahman	0.79	12.94	0.06 ± 0.01
AFC	Tabapua	1.82	21.38	0.08 ± 0.02
AFC	Multi‐breed	1.03	19.17	0.05 ± 0.003
ACP	Nellore	70.93	646.27	0.10 ± 0.01
ACP	Guzerat	59.73	527.77	0.10 ± 0.03
ACP	Brahman	93.61	440.47	0.18 ± 0.02
ACP	Tabapua	88.63	491.32	0.15 ± 0.05
ACP	Multi‐breed	120.60	584.70	0.17 ± 0.01

*Note:* σ^2^a: additive genetic variance; σ^2^e: residual variance; h^2^: heritability; SD: sampling standard deviation of h^2^ reported by BLUPF90+ from 10 000 samples. Heritability estimates are reported to two decimal places. Sampling standard deviations below 0.01 are reported to three decimal places.

Based on preliminary results, four approaches were tested for genomic evaluation of Nellore, Guzerat, Brahman, and Tabapua breeds:

- Single breed (G_SB_): This approach applied the ssGBLUP model separately within each breed, enabling direct comparison with multi‐breed methodologies.
- Multi‐breed using current allele frequencies (G_0_): This method utilized the traditional covariance matrix formulation of ssGBLUP (Legarra et al. 2009), with the inverse matrix defined as described by Aguilar et al. (2010):

(2) $$H^{-1}=A^{-1}+\left[\begin{array}{cc} 0 & 0\\ 0 & G^{-1}-A_{22}^{-1} \end{array}\right]$$

where **G** was built as VanRaden (2008), $G=ZZ^{\prime }$,

(3) $$Z=\left(M-P\right)/{\left[2\sum_{j=1}^{n}p_{j}\;\left(1-p_{j}\right)\right]}^{1/2}$$

where ***M*** is the matrix of *n* SNP genotypes for each animal, with elements coded as 0, 1, or 2, representing the count of the second allele for homozygotes, heterozygotes, and the alternative homozygotes, respectively. ***P*** is the matrix of twice the allele frequencies of the second allele at each locus.

iii Multi‐breed with the genomic relationship matrix (**G**) centered and scaled by breed‐specific allele frequencies, referred to as AGR. This matrix is constructed following Lourenco et al. (2016). Considering the differences in allele frequencies among breeds, the **Z** matrix for each breed is defined as follows:

(4) $$Z=\left(M-P_{K}\right)/{\left[2\sum_{j=1}^{n}p_{\mathrm{jK}}\;\left(1-p_{\mathrm{jK}}\right)\right]}^{1/2}$$

where each element of $P_{K}$ is $p_{\mathrm{jK}}$, and $p_{\mathrm{jK}}$ is the frequency of the second allele at locus *j* specific to breed *K*.

iv Multi‐breed with four metafounders representing the Nellore, Guzerat, Brahman, and Tabapua breeds (MF): The metafounders approach models the additive relationship matrix **A**(**Γ**), given a positive definite matrix defined as follows:
$$\Gamma =\left(\begin{array}{cccc} \gamma^{A} & \gamma^{A,B} & \gamma^{A,C} & \gamma^{A,D}\\ \gamma^{A,B} & \gamma^{,B} & \gamma^{B,C} & \gamma^{B,D}\\ \gamma^{A,C} & \gamma^{B,C} & \gamma^{C} & \gamma^{C,D}\\ \gamma^{A,D} & \gamma^{B,C} & \gamma^{C,D} & \gamma^{D} \end{array}\right).$$

where $\gamma^{A}$, $\gamma^{B}$, $\gamma^{C}$, and $\gamma^{D}$ represent the relationships within each metafounder, and $\gamma^{A,B}$, $\gamma^{A,C}$, $\gamma^{A,D}$, $\gamma^{B,C}$, $\gamma^{B,D}$, and $\gamma^{C,D}$ denotes the relationship coefficient between pairs of metafounders. All coefficients were estimated using combined genomic and pedigree information. The generalized least squares (GLS) approach for multiple populations, as proposed by Garcia‐Baccino et al. (2017), was applied using the GAMMAF90 program from the BLUPF90 suite (Lourenco et al. 2022; Misztal et al. 2014).

The inverse of the **H** matrix incorporating metafounders was calculated with the BLUP90IOD03 program, also part of the BLUPF90 family (Lourenco et al. 2022; Misztal et al. 2014):

(5) $${\left(H^{\Gamma }\right)}^{-1}={\left(A^{\Gamma }\right)}^{-1}+\left[\begin{array}{cc} 0 & 0\\ 0 & G_{05}^{-1}-{\left(A_{22}^{\Gamma }\right)}^{-1} \end{array}\right]$$

where $A^{\Gamma }$ and $A_{22}^{\Gamma }$ correspond to the pedigree relationship $A^{-1}$ and $A_{22}^{-1}$ modified by a positive definite matrix, and $G_{05}^{-1}$ is the inverse of the genomic relationship matrix for genotyped animals assuming allele frequencies equal to 0.5.

All evaluated approaches are summarized in Table 3. Accordingly, different covariance structures among animals were considered in the construction of the **G** for the analyzed populations.

**TABLE 3:** Scenarios in the multi‐breed evaluation of Nellore, Guzerat, Brahman, and Tabapua population in Brazil.

Scenario	Description
**G**_SB_	ssGBLUP performed separately for each breed
**G**_0_	Multi‐breed model with standard **G** matrix constructed using current allele frequencies without adjustments, following VanRaden (2008)
AGR	Multi‐breed model with **G** matrix centered and scaled by breed‐specific allele frequencies, as described by Lourenco et al. (2016)
MF	Multi‐breed model with four metafounders: Nellore, Guzerat, Brahman, and Tabapua

### 2.5 Population Structure and Genomic Diversity

The principal component analysis (PCA) was performed using genotypic data with the preGSF90 software from the BLUPF90 suite (Aguilar et al. 2014), considering the current allele frequencies estimated for the whole population (G_0_ scenario). The objective was to investigate population structure and assess genomic relationships among the Zebu breeds included in the study. The genotype matrix was derived from a quality‐controlled multi‐breed SNP panel, and eigenvalue decomposition was carried out based on the first two principal components to visualize the genetic dispersion among individuals. Two analyses were performed: the first included all four breeds (Nellore, Guzerat, Brahman, and Tabapua), and the second included only Guzerat, Brahman, and Tabapua. These analyses allowed the elucidation of specific patterns of genetic structure both within and across the evaluated populations. Pairwise genetic differentiation among breeds was quantified in PLINK 2.0 (Chang et al. 2015) using the Hudson *F*_ST_ estimator and the ratio‐of‐averages approach across SNPs retained after quality control (Hudson et al. 1992; Bhatia et al. 2013).

### 2.6 Validation and Scenarios Comparison

The linear regression (LR) method (Legarra and Reverter 2018) was employed to evaluate the performance of the different scenarios in predicting genomic estimated breeding values (GEBV) by assessing accuracy, bias, and dispersion parameters. These statistics were computed using the validationf90 software (Bermann et al. 2025), which calculates validation metrics and their respective confidence intervals.

This evaluation involved comparing two different datasets: the “complete” dataset, which included all available phenotypic information to calculate the full GEBV (${\hat{u}}_{w}$); and the “partial” dataset, which excluded phenotypic records of animals born in 2021 to calculate the partial GEBV (${\hat{u}}_{p}$). Validation was performed using young animals with both genotypes and phenotypes available, with the cut‐off depending on the trait: for carcass traits (REA and RFT), animals born in 2021; for AFC, animals born in 2020; and for ACP, animals born in 2019 (Table 4). This strategy mimics practical genomic selection scenarios, in which the GEBV of young selection candidates is predicted using information from older, proven animals. Pedigree and genomic links were retained in the partial evaluation, whereas the focal animals' own phenotypes were removed. Therefore, parents, full siblings, half siblings, and more distant relatives could contribute information when present, as occurs in routine genetic evaluation. The LR method evaluates population‐level accuracy, bias, and dispersion under this available relationship structure rather than prediction in a genetically disconnected validation set.

**TABLE 4:** Number of records in the complete and partial datasets and validation animals used in the LR method to evaluate the statistics of the different scenarios for rib eye area (REA), rump fat thickness (RFT), age at first calving (AFC), and accumulated productivity (ACP).

Trait		Breed				Total
		Nellore	Guzerat	Brahman	Tabapua	
REA (cm^2^)	Complete	150 992	5011	4086	1648	161 737
	Partial	149 691	4497	3762	1394	159 344
	Validation	1301	514	324	254	2393
RFT (mm)	Complete	150 643	5011	4086	1648	161 388
	Partial	149 342	4497	3762	1394	158 995
	Validation	1301	514	324	254	2393
AFC (months)	Complete	176 607	12 555	38 075	7779	235 016
	Partial	174 448	12 175	36 773	7699	231 095
	Validation	2159	380	1302	80	3921
ACP (kg)	Complete	64 980	4734	18 483	2033	90 230
	Partial	63 789	4504	17 618	1985	87 896
	Validation	1191	230	865	48	2334

Prediction accuracy was estimated based on the covariance between the GEBVs obtained from the complete dataset ${\hat{u}}_{w}$ and the partial dataset ${\hat{u}}_{p}$, adjusted by the average inbreeding coefficient $\bar{F}$ of the validation animals, and the estimated additive genetic variance $\hat{\sigma_{u}^{2}}$, as follows:

(6) $$\hat{\mathrm{acc}}=\sqrt{\frac{\mathrm{cov}\left({\hat{u}}_{w},{\hat{u}}_{p}\right)}{\left(1-\bar{F}\right)\hat{\sigma_{u}^{2}}}}$$

The bias was estimated based on the difference between the means of predicted genetic values in each set, standardized by the root of genetic variance:

(7) $$\mu w_{P}=\frac{\bar{\hat{u}}p-\bar{\hat{u}}w}{\sigma_{u}}$$

The dispersion was calculated as the linear regression coefficient between the GEBV obtained with the complete and partial datasets, as follows:

(8) $$b_{w,p}=\frac{\mathrm{cov}\left({û}_{w},{û}_{p}\right)}{\mathrm{var}\left({û}_{p}\right)}$$

## 3 Results

### 3.1 Population Structure

**FIGURE 1:**
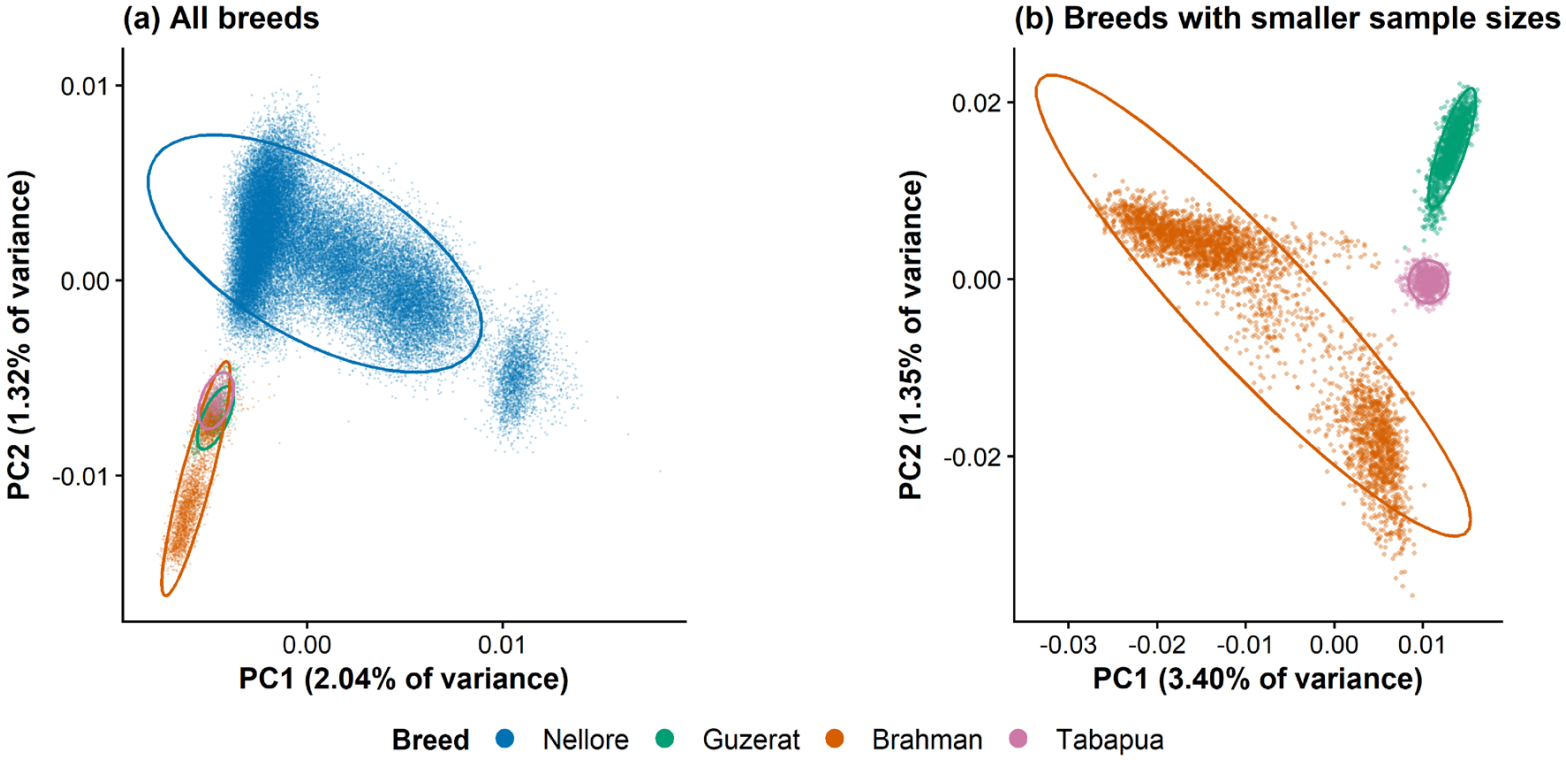
Principal component analysis (PCA) showing genetic structure among four Zebu cattle breeds. Nellore (blue), Guzerat (green), Brahman (orange), and Tabapua (purple). (a) All breeds. (b) Breeds with smaller sample sizes. Each point represents one genotyped animal; ellipses are 95% probability ellipses based on a multivariate t distribution.

The PCA (Figure 1) showed broad overlap among the four breeds, together with partial differentiation in specific comparisons. Although the distribution suggests some differentiation between Nellore and the other Zebu breeds, the points representing Guzerat, Brahman, and Tabapua showed substantial overlap, indicating limited genetic divergence in the first two principal components. PC1 and PC2 accounted for 2.04% and 1.32% of the total variance, respectively, when all breeds were included, and 3.40% and 1.35% when only Guzerat, Brahman, and Tabapua were considered. The slight dispersion observed in Nellore likely reflects its larger sample size and greater within‐breed genetic diversity, rather than distinct separation from the other breeds. In panel B, Brahman showed visible differentiation along PC1, although overlap with Guzerat and Tabapua remained. Because the first two components explained a small proportion of total variation, this pattern indicates partial rather than complete separation. These findings align with Londoño‐Gil et al. (2025), who reported minimal divergence among Zebu breeds based on principal component analysis. Notably, the present study used a larger number of genotyped animals for the smaller breeds, which enhances the reliability of these observations and supports the potential for joint genomic evaluations in multi‐breed contexts.

Pairwise Hudson *F*_ST_ estimates ranged from 0.033 to 0.047 (Table 5), indicating low but detectable genetic differentiation among breeds. The lowest estimate was observed between Brahman and Guzerat (0.033), whereas the highest was between Nellore and Tabapua (0.047). These results complement the PCA by showing that the partial separation observed along the first principal components occurred against a background of substantial shared genomic variation among breeds.

**TABLE 5:** Pairwise Hudson *F*_ST_ estimates among Nellore, Guzerat, Brahman, and Tabapua breeds.

Breed	Nellore	Guzerat	Brahman	Tabapua
Nellore	0	0.045	0.040	0.047
Guzerat	0.045	0	0.033	0.038
Brahman	0.040	0.033	0	0.038
Tabapua	0.047	0.038	0.038	0

*Note:* *F*_ST_: fixation index. Estimates were calculated in PLINK 2.0 using the Hudson method and the ratio‐of‐averages approach.

The **Γ** matrix, constructed based on the metafounders of the four breeds, indicated moderate genomic relationships among them. The diagonal elements, which represent relationships within each base population, ranged from 0.68 (Guzerat) to 0.77 (Brahman). The off‐diagonal elements, reflecting relationships between breeds, varied from 0.66 to 0.69, suggesting relatively balanced and homogeneous genetic connections among these Zebu populations. These findings support the feasibility of joint evaluations using the metafounder approach, and aligning with the expected ancestral proximity among these indicine breeds. Furthermore, they indicate a significant potential for genetic sharing across breeds and reinforce the suitability of applying a multi‐breed evaluation strategy with metafounders, as proposed by Legarra et al. (2015).

$$\Gamma ={\left[\begin{array}{ccc} 0.71\; & 0.66\; & 0.68\;\;0.67\\ 0.66\; & 0.68\;\; & \begin{array}{cc} 0.68\; & 0.66 \end{array}\\ \begin{array}{c} 0.68\;\\ 0.67\; \end{array} & \begin{array}{c} 0.68\;\\ 0.66\; \end{array} & \begin{array}{c} \begin{array}{cc} 0.77\; & 0.69 \end{array}\\ \begin{array}{cc} 0.69\; & 0.72 \end{array} \end{array} \end{array}\right]}_{4x4}$$

### 3.2 Validation Statistics

**FIGURE 2:**
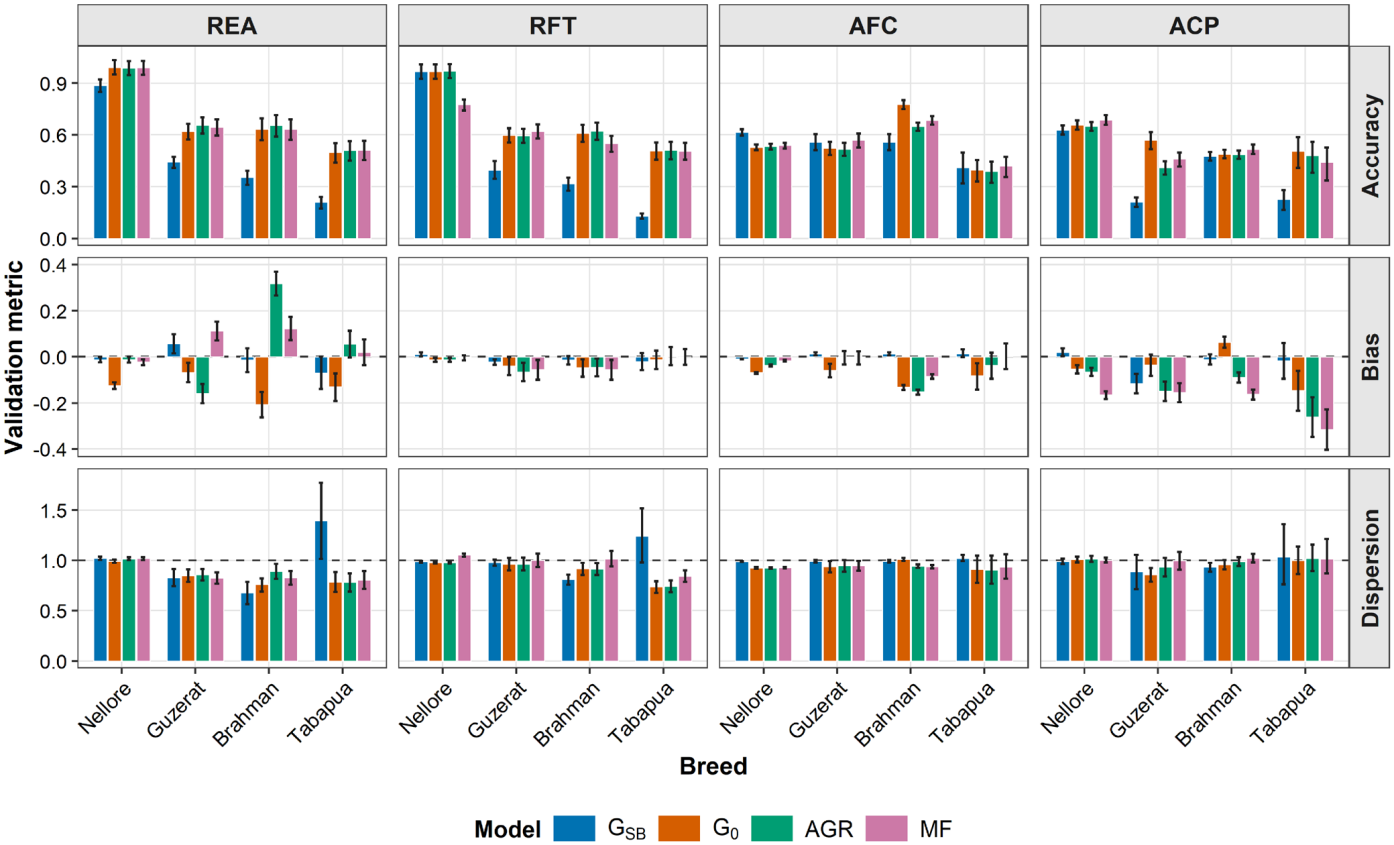
Accuracy, bias (scaled by the genetic standard deviation), and dispersion of genomic predictions for validation animals across traits and breeds. Bars represent estimates, and error bars indicate the corresponding 95% confidence intervals obtained with validationf90. Traits: REA, rib eye area; RFT, rump fat thickness; AFC, age at first calving; and ACP, accumulated productivity. Models: G_SB_, single‐breed model; G_0_, multi‐breed model with common allele frequencies; AGR, multi‐breed model with breed‐specific allele frequencies; and MF, metafounders model accounting for ancestral population structure.

Figure 2 presents the validation results of genomic predictions for the four evaluated traits (REA, RFT, AFC, and ACP) by breed and model. In general, the relative performance of the multi‐breed and GSB models varied across breeds and traits, including those with smaller sample sizes, such as Guzerat, Brahman, and Tabapua.

### 3.3 Genetic Trends

**FIGURE 3:**
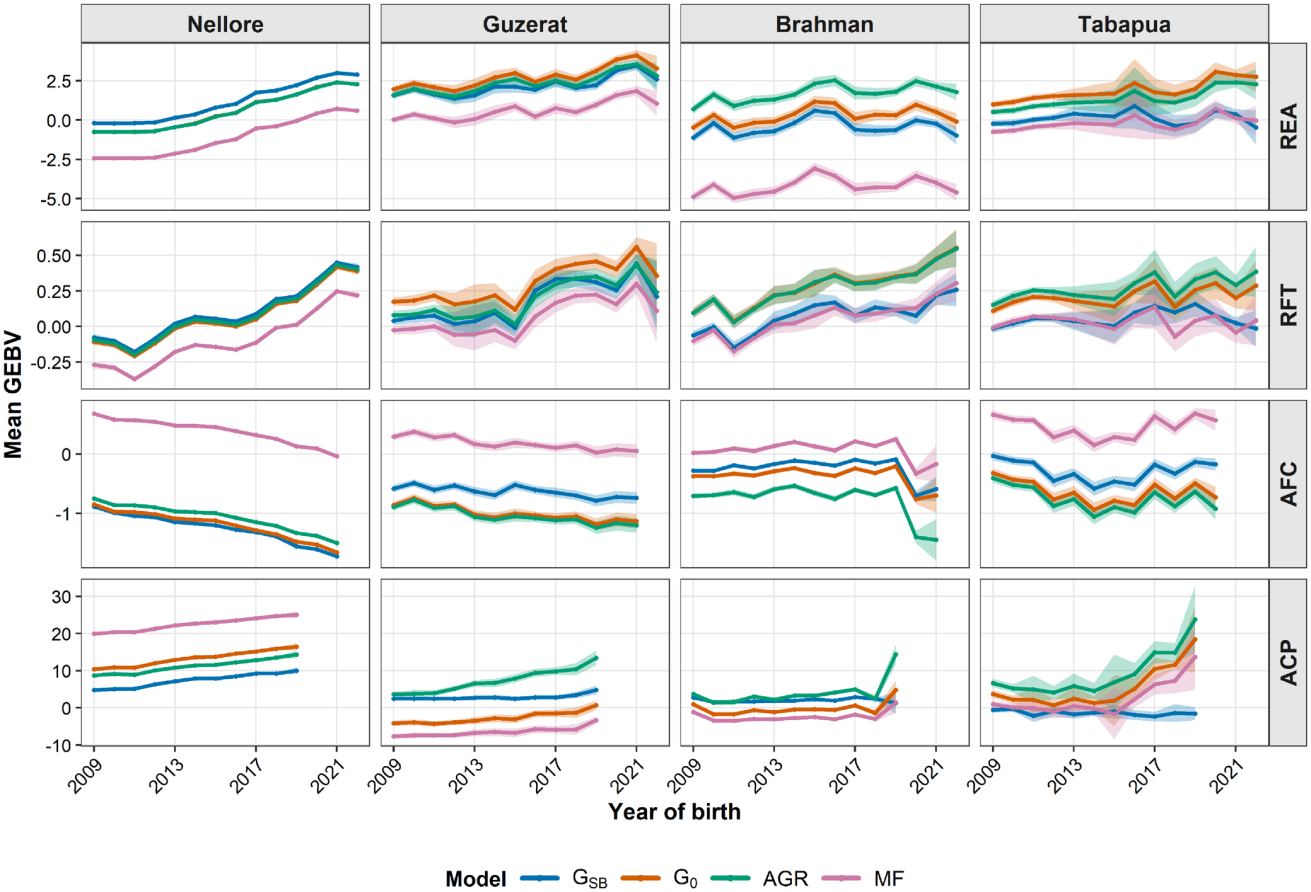
Genetic trends in mean genomic estimated breeding values (GEBV) by breed and prediction model for rib eye area (REA), rump fat thickness (RFT), age at first calving (AFC), and accumulated productivity (ACP), from 2009 to 2021. Models: G_SB_, single‐breed; G_0_, multi‐breed with common allele frequencies; AGR, breed‐specific allele frequencies; and MF, metafounder model. Shaded areas represent 95% confidence intervals of the annual mean (mean ± 1.96 SE). ACP trends are restricted to 2009–2019 because of the latency of phenotypic expression.

Genetic trends differed among traits, breeds, and prediction models (Figure 3). For REA, all methodologies indicated positive genetic progress over time, although the magnitude of the trend was consistently smaller under MF. AGR generated steeper RFT trends in Brahman and Tabapua, whereas MF produced smoother trajectories. AFC declined under all models, more gradually under MF, and Nellore showed the most consistent ACP trends under MF. Overall, GSB fluctuated more across traits and breeds than MF and AGR.

## 4 Discussion

The performance of the models was evaluated by grouping results by breed, allowing identification of breed‐specific response patterns to the multi‐breed methodologies (MF, AGR, and G_0_) compared with the single‐breed model (G_SB_). Comparisons were based on 95% confidence intervals (95% CI) to avoid over interpretation of numerical differences and to focus the discussion on statistically supported gains, as recommended by Bermann et al. (2025).

In breeds with limited phenotypic and genotypic data, multi‐breed models produced higher accuracy than GSB in several analyses, although not all differences were statistically supported. This finding supports the hypothesis that information sharing through common ancestry increases prediction accuracy and calibration in underrepresented populations (Hayes et al. 2009; Raymond et al. 2018).

For the Brahman breed, the advantages of the multi‐breed approach were evident across all evaluated traits. For example, for rib eye area (REA), the metafounder (MF) model achieved an accuracy of 0.63, statistically outperforming the G_SB_ model, which showed an accuracy of 0.35. The lack of confidence interval overlap confirmed that these gains were statistically significant. In addition to improved accuracy, multi‐breed models exhibited better results, with dispersion values closer to unity, whereas G_SB_ showed substantial deviations.

For age at first calving (AFC), the multi‐breed models also outperformed the single‐breed approach. The AGR model yielded high accuracy, while MF showed consistent results, outperforming G_SB_ in scenarios where the single‐breed model showed lower predictive accuracy. These findings align with those of Christensen (2012) and Kluska et al. (2021), who observed that single‐breed models tend to be less robust in small populations due to limited connectedness among individuals, a limitation mitigated by metafounders or AGR matrices.

For the Guzerat breed, observed improvements were not merely numerical. For REA, the MF model achieved an accuracy of 0.64, significantly higher than G_SB_ at 0.44, with non‐overlapping confidence intervals ranging from 0.59 to 0.69 for MF and from 0.40 to 0.47 for G_SB_. This increase in predictive accuracy is particularly relevant for carcass traits, which are costly to measure and typically have limited phenotypic records (Gordo et al. 2012). For AFC, the AGR model had the highest numerical accuracy in the Guzerat breed. However, overlapping confidence intervals with MF and G_0_ indicated no statistically significant differences among multi‐breed models. The stability of estimates from joint analyses underscores the importance of using genetically related breeds, such as Nellore, to strengthen predictions in smaller populations (Cesarani et al. 2022).

For the Tabapua breed, multi‐breed modeling did not consistently result in statistically significant increases in accuracy, as indicated by overlapping confidence intervals. Instead, its main contribution was an improvement in the scaling of genomic predictions, as reflected by dispersion values closer to unity. For REA, the G_SB_ model showed a dispersion of 1.39, whereas the MF model reduced this value to 0.81, indicating better agreement between predicted and observed genetic variability. For RFT, a dual benefit was observed, with the MF model increasing accuracy from 0.13 under G_SB_ to 0.51 and simultaneously reducing dispersion from 1.24 to 0.84, with non‐overlapping confidence intervals confirming the statistical relevance of these gains.

In contrast, for accumulated productivity (ACP), all models displayed high dispersion values, indicating that even multi‐breed approaches remain constrained by data structure. The limited number of females with concurrent phenotypic and genotypic records reduces population connectedness and restricts precise parameter estimation for this trait (Legarra et al. 2015; Lourenco et al. 2020). Furthermore, ACP is a long‐term composite index that naturally accumulates environmental noise over successive reproductive years. This introduces residual variability that makes it a particularly challenging target for genomic modeling, regardless of the evaluation strategy.

In the Nellore breed, which has the most robust data structure and serves as an anchor population for multi‐breed analyses, differences among methodologies were attenuated. Specifically, for REA, RFT, and AFC, multi‐breed models and G_SB_ showed statistically equivalent performance, with substantial overlap in confidence intervals. In contrast, for ACP, the MF model achieved higher numerical accuracy at 0.69 compared with 0.48 for G_SB_, while differences relative to G_0_ were small and not statistically supported. Overall, these findings indicate that the benefits of multi‐breed modeling tend to diminish as within‐breed data density increases, although improvements in calibration may still be observed even in well‐represented populations (Macedo et al. 2022; Jang et al. 2022).

Dispersion values between 0.95 and 1.05 were considered indicative of adequately calibrated genomic predictions (Mäntysaari et al. 2010; Patry and Ducrocq 2011) and were more frequent in the MF and G_0_ models. By contrast, G_SB_ often exceeded acceptable limits, especially for reproductive traits. The bias estimates reinforced this pattern, with MF and G_0_ maintaining stability for most traits. In the Tabapua breed, high bias and dispersion occurred together, suggesting that multi‐breed approaches require a minimum level of data structure and population connectedness to generate reliable predictions (Legarra et al. 2015; Kluska et al. 2021).

### 4.1 Relevance of the Genetic Trends

Beyond point‐based validation metrics, genetic trends provide a complementary diagnostic of the temporal scale and stability of genomic predictions. They do not constitute an independent measure of predictive accuracy and should therefore be interpreted together with the LR validation statistics.

The more conservative REA trajectory under MF may indicate reduced inflation of genetic gain and improved modeling of the base population structure, as reported by Macedo et al. (2022) and Londoño‐Gil et al. (2025).

The steeper RFT trends under AGR may reflect greater sensitivity to differences in ancestral allele frequencies and base population composition (Kudinov et al. 2020). In contrast, the smoother trajectories under MF are consistent with its dispersion performance in the validation analysis.

For AFC, the more gradual decline under MF may indicate reduced overestimation of genetic merit and improved temporal stability, consistent with Meyer et al. (2018). For ACP, the consistency observed in Nellore highlights the importance of a well‐structured and densely connected reference population for stable long‐term predictions (Kluska et al. 2021; Belay et al. 2025).

Taken together, the genetic trends complement the validation results by showing how model‐specific predictions behave over time. Their greater stability under some multi‐breed models is particularly relevant for traits and populations with limited data availability (Xu, Wang, et al. 2019; Xu, Zhu, et al. 2019; Misztal et al. 2021).

The marked imbalance in breed representation requires cautious interpretation. Nellore contributed most phenotypes and genotypes and therefore acted as the anchor population in the joint evaluation. Gains in Guzerat, Brahman, and Tabapua may consequently reflect information borrowed mainly from Nellore rather than balanced exchange among breeds. In addition, the small validation sets for some breed and trait combinations, particularly Tabapua ACP, reduced the power to distinguish models. These results should not be generalized to balanced or weakly connected populations without further evaluation.

Differences between carcass and reproductive traits may reflect both recording structure and biology. REA and RFT can be measured in males and females at a relatively young age under standardized ultrasound protocols. AFC and ACP are female‐limited, and ACP is expressed over a longer period and combines reproductive, maternal, longevity, and management components. Reproductive traits also have lower heritability and greater environmental sensitivity, which can reduce the transferability of genomic information across herds and breeds. The variance‐component estimates are therefore important for interpreting differences in predictive performance.

This interpretation is consistent with studies of genomic prediction in *Bos indicus* and tropical beef cattle showing that reference‐population size, connectedness, and the treatment of ancestral relationships influence predictive performance (Kluska et al. 2021; Rodriguez Neira et al. 2022; Londoño‐Gil et al. 2025).

In summary, the AGR and MF models showed advantages over the single‐breed approach in specific data‐limited scenarios for carcass and reproductive indicator traits, displaying higher prediction accuracy and more robust predictions. However, for the Nellore breed, differences among methodologies were minor. This contrast reinforces that the effectiveness of multi‐breed models strongly depends on population structure and data density. Overall, these findings are consistent with previous studies emphasizing the need for careful application of multi‐breed approaches, coupled with continuous enrichment of reference populations, to maximize genetic gain while minimizing bias in genomic evaluations (Cesarani et al. 2021; Melo et al. 2024; Londoño‐Gil et al. 2025).

The main limitations of this study were the marked imbalance in breed representation, the small validation groups for some breed and trait combinations, the retention of relatives in the routine‐evaluation validation design, and the use of PCA and pairwise F_ST_ as summary measures of population structure. Continued collection of phenotypes and genotypes in the less‐represented breeds remains necessary.

## 5 Conclusion

Multi‐breed evaluation enhanced genomic prediction accuracy in several breed–trait combinations with limited data availability, whereas gains were generally smaller in Nellore populations. Among the evaluated approaches, the metafounder model showed favorable performance across multiple scenarios; however, no single multi‐breed method consistently outperformed the others. The effectiveness of each approach depended on the structure of the reference population, the trait analyzed, and the amount of available phenotypic and genomic information. Therefore, multi‐breed evaluation should be viewed as a complementary strategy to increase prediction accuracy, particularly for underrepresented populations, rather than a substitute for expanding phenotypic and genomic reference datasets.

## Author Contributions

**Matias Bermann:** conceptualization, methodology, software, writing – review and editing, validation, visualization. **Fernando Baldi:** conceptualization, investigation, writing – original draft, methodology, validation, visualization, writing – review and editing, formal analysis, supervision, data curation, project administration, resources. **Larissa Temp:** validation, methodology. **Miller Teodoro:** conceptualization, visualization, writing – original draft. **Ignacio Aguilar:** conceptualization, writing – review and editing, validation, methodology, software. **Daniela Lourenco:** validation, visualization, software, methodology, supervision. **Angelica S. C. Pereira:** visualization, writing – original draft, writing – review and editing. **Gabriel Gubiani:** investigation, methodology, writing – original draft, visualization, validation, formal analysis. **Eduarda Oliveira:** writing – original draft. **Marisol Londoño‐Gil:** conceptualization, methodology, visualization, supervision.

## Funding

This work was supported by the Fundação de Amparo à Pesquisa do Estado de São Paulo (2023/03659‐5).

## Conflicts of Interest

The authors declare no conflicts of interest.

## Data Availability

The data that support the findings of this study are available from National Association of Breeders and Researchers—ANCP. Restrictions apply to the availability of these data, which were used under license for this study. Data are available from https://www.ancp.org.br/ with the permission of National Association of Breeders and Researchers—ANCP.
